# Cysteine Surface Engineering of Green-Synthesized Gold Nanoparticles for Enhanced Antimicrobial and Antifungal Activity

**DOI:** 10.3390/ijms26157645

**Published:** 2025-08-07

**Authors:** Karen M. Soto, Angelica Gódinez-Oviedo, Adriana Romo-Pérez, Sandra Mendoza, José Mauricio López-Romero, Gerardo Torres-Delgado, Jorge Pineda-Piñón, Luis M. Apátiga-Castro, José de Jesús Pérez Bueno, Alejandro Manzano-Ramírez

**Affiliations:** 1 Centro de Investigaciones y de Estudios Avanzados del I.P.N., Unidad Querétaro, Querétaro 76230, Mexico; jm.lopez@cinvestav.mx (J.M.L.-R.); gtorres@cinvestav.mx (G.T.-D.); 2 Departamento de Investigación y Posgrado en Alimentos, Facultad de Química, Universidad Autónoma de Querétaro, Querétaro 76010, Mexico; qa_angelica@hotmail.com (A.G.-O.); smendoza@uaq.mx (S.M.); 3 Instituto de Química, Universidad Nacional Autónoma de México, Circuito Exterior s/n, Ciudad Universitaria, C.P., Coyoacán, Ciudad de Mexico 04510, Mexico; adriana.romo@iquimica.unam.mx; 4 Centro de Investigación en Ciencia Aplicada y Tecnología Avanzada, Instituto Politécnico Nacional, Unidad Querétaro, Cerro Blanco No. 141, Colinas del Cimatario, Querétaro 76090, Mexico; jpinedap@ipn.mx; 5 Centro de Física Aplicada y Tecnología Avanzada (CFATA), Universidad Nacional Autónoma de México, A.P. 1-1010, Querétaro 76230, Mexico; apatiga@unam.mx; 6 Centro de Investigación y Desarrollo Tecnológico en Electroquímica, S.C., Parque Tecnológico, Querétaro Sanfandila, Pedro Escobedo, Santiago de Querétaro 76703, Mexico; jperez@cideteq.mx

**Keywords:** gold nanoparticles, green synthesis, cysteine functionalization, *Schinus molle*

## Abstract

Green synthesis of gold nanoparticles (AuNPs) provides a significantly eco-friendly and low-impact counterpart to conventional chemical methods. In the present study, we synthesized gold nanoparticles using *Schinus molle* (P-AuNPs) aqueous extract as a reducing and stabilizing agent. The obtained nanoparticles were then stabilized by another biocompatible agent, the chiral amino acids L-cysteine (L-Cys-AuNPs) and D-cysteine (D-Cys-AuNPs), to estimate the potential of the surface modification for enhancing AuNPs surface chemistry and antimicrobial action. The synthesized gold nanoparticles were confirmed by UV-Vis spectroscopy, FTIR, XRD, and circular dichroism to validate their formation, crystalline structure, surface properties, and chirality. Physicochemical characterization confirmed the formation of crystalline AuNPs with size and morphology modulated by chiral functionalization. TEM and DLS analyses showed that L-cysteine-functionalized AuNPs were smaller and more uniform, while FTIR and circular dichroism spectroscopy confirmed surface binding and the induction of optical activity, respectively. L-Cys-AuNPs exhibited the highest antimicrobial efficacy against a broad spectrum of microorganisms, including *Escherichia coli*, *Salmonella enterica*, *Listeria monocytogenes*, *Staphylococcus aureus*, *Staphylococcus epidermidis*, and, notably, *Candida albicans*. L-Cys-AuNPs showed the lowest MIC and MBC values, highlighting the synergistic effect of chirality on biological performance. These findings suggest that L-cysteine surface engineering significantly enhances the therapeutic potential of AuNPs, particularly in combating drug-resistant fungal pathogens such as *C. albicans*. This research paves the way for the development of next-generation antimicrobial agents, reinforcing the relevance of green nanotechnology in the field of materials science and nanotechnology.

## 1. Introduction

Gold nanoparticles (AuNPs) have emerged as versatile nanomaterials due to their unique physicochemical properties, such as localized surface plasmon resonance, high surface-area-to-volume ratio, and ease of surface functionalization. Beyond their well-established roles in imaging, diagnostics, and catalysis, recent research has explored their potential in chiral nanotechnology and biomedical applications, including antimicrobial activity [1,2]. The primary method of synthesis is the chemical reduction of Au^3+^ ions in solution, typically mediated by reductants such as NaBH_4,_ which results in toxic residues that pose risks to ambient and human health. To eliminate the generation of toxic waste, green synthesis emerges as a viable option that can be carried out using plants and microorganisms. The use of plant extracts for the synthesis of AuNPs is an important alternative to reduce the Au^3+^ ions to Au^0^, mediated by OH^−^ groups present in different secondary metabolites such as carbohydrates, polyphenols, flavanols, phenolic acids, carotenoids, terpenoids, and proteins, which are the principal components in plant extracts. After the “reduction phase”, the small nuclei of Au^0^ ions are grouped, and, finally, the oxidized secondary metabolites cap the surface of the AuNPs, resulting in the stabilization of the NPs [1,2]. *Schinus molle* L. (Pirul) can be used for the green synthesis of AuNPs due to the presence of secondary metabolites. Pirul is a tree native to South and Central America, belonging to the *Anacardiaceae* family. Their fruit is a berry commonly known as pink pepper or American pepper. It has been used in traditional medicine as an analgesic, for respiratory and urinary infections, digestive and purgative diuretic, for toothache, and against rheumatism and menstrual disorders due to its antibacterial, antiviral, topical antiseptic, antifungal, antioxidant, anti-inflammatory, anti-tumoral, antispasmodic, and analgesic properties, related to the presence of different phenolic compounds like gallic acid, catechin, vanillic acid, and coumarin, among others [3,4,5]. Previously *Schinus molle* have been used for the synthesis of metallic nanoparticles such as gold [6] and silver [7], successfully obtaining nanoparticles with different morphologies and sizes, but its antimicrobial and antifungal activity have not yet been explored.

The induction of chirality on the surface of gold nanoparticles (AuNPs) represents a significant advancement in the design of nanomaterials, particularly in the context of biomedical applications. Surface chirality can profoundly influence the interaction between nanoparticles and biological systems, as many biomolecules and cellular processes are inherently chiral [8,9]. By functionalizing AuNPs with enantiomerically pure molecules such as L- or D-cysteine, it is possible to modulate not only the physicochemical characteristics of the nanoparticles, including size, morphology, surface charge, and optical activity, but also their biological behavior [10,11]. Chiral gold nanoparticles (chiral AuNPs) exhibit optical activity, typically observed through circular dichroism (CD) spectroscopy, making them promising candidates for enantioselective sensing, asymmetric catalysis, and chiroptical devices. Chirality in AuNPs can be introduced via various strategies, including the use of chiral ligands, templates, or through asymmetric growth processes that result in inherently chiral morphologies [12,13].

One effective strategy for imparting chirality to AuNPs involves surface modification with chiral molecules, such as amino acids. Cysteine, a naturally occurring chiral amino acid with a thiol group, is particularly well-suited for this purpose due to its strong affinity for gold surfaces. When cysteine binds to AuNPs, it can induce a chiral arrangement of surface atoms or influence the asymmetric growth of the nanoparticles, resulting in chiroptical activity detectable via circular dichroism (CD) spectroscopy [14,15].

In addition to their chiral properties, cysteine-functionalized AuNPs have shown promising antimicrobial activity. The combination of gold’s intrinsic stability and bioinert nature with the reactive functional groups of cysteine enhances interactions with microbial membranes, often leading to oxidative stress, membrane disruption, and inhibition of cellular functions. Studies have demonstrated that these nanostructures can be effective against a broad spectrum of pathogens, including Gram-positive and Gram-negative bacteria, with potential for overcoming antibiotic resistance mechanisms. The integration of chiral modulation and antimicrobial functionality in cysteine-modified AuNPs positions them as powerful tools in the development of smart, multifunctional nanoplatforms for biomedical applications such as targeted drug delivery, biosensing, and next-generation antimicrobial agents [16].

The present study aims to evaluate the impact of cysteine surface modification on green AuNPs synthesized using an aqueous extract of *Schinus molle* fruit. Specifically, the objective is to investigate how this chiral functionalization influences the nanoparticles’ morphology, size, crystallinity, and, most importantly, their antimicrobial and antifungal activity. The potential applications of this study are vast, as it could lead to the synthesis of chiral materials with applications in antimicrobial and antifungal treatments. We present the first comparative evaluation of L- and D-cysteine surface modification in green-synthesized AuNPs using this extract, coupled with their antifungal efficacy against *Candida albicans*. This dual approach offers new insights into chirality-induced antimicrobial enhancement using sustainable methodologies.

## 2. Results

### 2.1. Antioxidant Activity and Total Phenolic Content of Schinus Molle Extract

**Figure 1:**
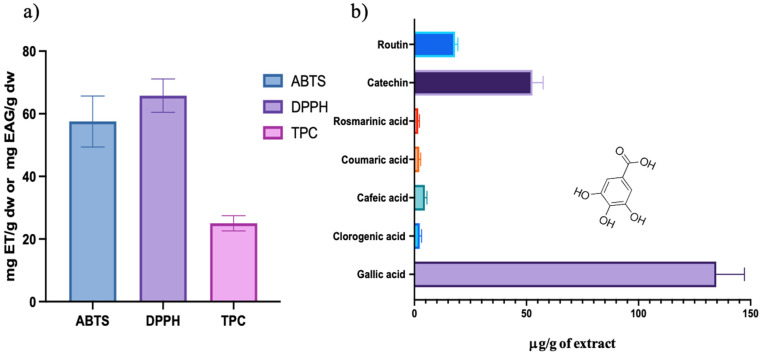
(**a**) Antioxidant activity of *Schinus molle* L. aqueous extract and (**b**) phenolic compound concentrations obtained by HPLC of *Schinus molle* L. aqueous extract.

The aqueous extraction yield from *Schinus molle* fruits was 2.85 ± 0.47%, reflecting an efficient recovery of water-soluble bioactive compounds. To evaluate the antioxidant activity of the *S. molle* extract, the DPPH (2,2-diphenyl-1-picrylhydrazyl) and ABTS·+ (2,2′-Azino-bis (3-ethylbenzothiazoline-6-sulfonic) acid) methods were employed. Both methods rely on free radical scavenging, where antioxidants donate electrons or hydrogen atoms to neutralize free radicals, thus reducing the absorbance of the radical solution, due to the formation of a stable reduced colored product. Figure 1a displays the antioxidant activity measured by DPPH and ABTS assays, as well as the total phenolic content (TPC). The extract exhibited considerable radical scavenging activity, with values of 65.79 ± 5.32 mg Trolox equivalents (TE)/g dry weight (dw) for DPPH and 57.53 ± 8.15 mg TE/g dw for ABTS. These results demonstrate the extract’s strong antioxidant potential and hydrogen-donating ability. The values are consistent with those reported for methanolic extracts and essential oils from *S. molle* leaves and fruits, confirming the significant antioxidant capacity of the aqueous extract [4,17,18,19].

The total phenolic content was determined to be 25.02 ± 2.42 mg gallic acid equivalents (GAE)/g dw, suggesting a strong correlation between antioxidant capacity and polyphenol content. HPLC analysis (Figure 1b) identified gallic acid (134.67 μg/g dw), catechin (52.64 μg/g dw), and rutin (18.12 μg/g dw) as the major phenolic constituents. Bvenura et al., in 2022, reported a similar phenolic profile in an aqueous extract of *S. molle* collected in Africa; the concentrations of the phenolic compounds they obtained are largely similar to the ones obtained in the plant collected in Mexico, but the treatment of the extracts was different, which explains the different concentrations [3].

Among these, gallic acid (GA) plays a particularly important role in the green synthesis of metallic nanoparticles, including copper [20], iron [21], silver [22], and gold [23]. Its effectiveness lies in its dual function as a reducing and capping agent. Structurally, the hydroxyl (-OH) groups in the ortho and para positions of the aromatic ring allow for the direct reduction of metal ions, while the carboxyl (-COOH) group contributes to electron donation and coordination with metal surfaces. These features facilitate the stabilization of nanoparticles in aqueous media by preventing agglomeration. Therefore, the high content of gallic acid and other polyphenols not only explains the extract’s strong antioxidant activity but also supports its successful application in the green synthesis of gold nanoparticles (AuNPs), which will be discussed in the following sections [23].

### 2.2. UV-Vis Spectra

The green synthesis of gold nanoparticles was confirmed by the change in color of the colloidal solution; the P-AuNPs present a wine-red color, and, after the functionalization of particles, the color changed to purple. The successful synthesis of AuNPs with the aqueous extract of *S. molle* is due to the high content of gallic acid, catechin, and rutin present in the extract, which supports its dual function in the green synthesis of AuNPs, acting as both a reducing agent (via electron-donating hydroxyl and carboxyl groups) and a capping agent (stabilizing nanoparticles by steric and electrostatic mechanisms). Compared to conventional chemical routes, this green synthesis offers advantages such as the absence of hazardous solvents, and the inherent biocompatibility of the phytochemicals involved. These benefits highlight the potential of *S. molle* extract for scalable and eco-friendly nanomaterial production. In past studies, we have compared the green synthesis of gold nanoparticles with plant extracts against a chemical synthesis with NaBH_4_, observing greater stability across time as well as smaller diameters and increments in the biological activity [24].

**Figure 2:**
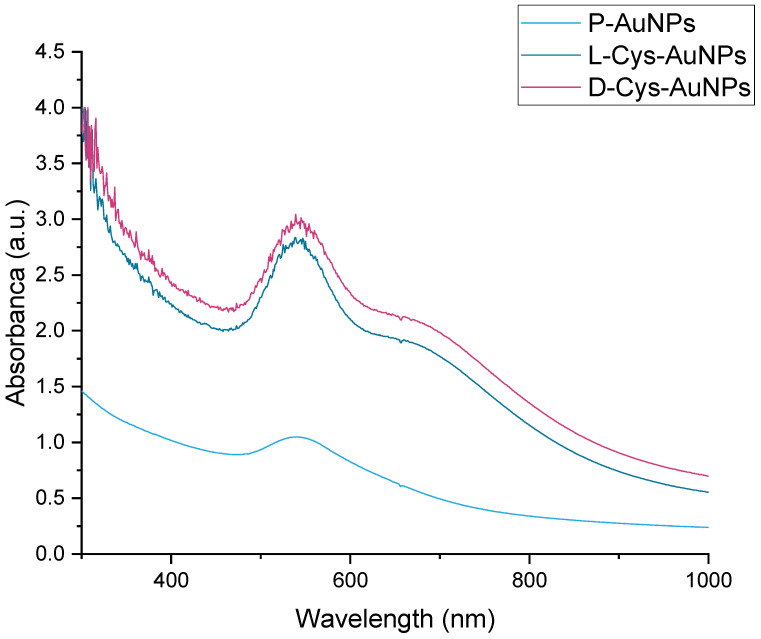
UV-Vis spectra of P-AuNPs, L-Cys-AuNPs, and D-Cys-AuNPs.

The resulting colloidal solutions were examined by UV-Vis spectroscopy. Figure 2 shows the optical properties of gold nanoparticles synthesized with Pirul extract (P-AuNPs) and functionalized with L-cysteine (L-Cys-AuNPs) and D-cysteine (D-Cys-AuNPs). All spectrums exhibit an absorption maximum at 538–539 nm; this peak corresponds to the surface plasmon resonance (SPR) band characteristic of the AuNPs, confirming the successful synthesis in all cases. Some minimal differences are observed in the absorbance intensity and morphology of the band. For example, P-AuNPs (light blue) shows a slightly narrower and less intense SPR peak, which generally indicates smaller particles or less aggregation, while L-Cys-AuNPs and D-Cys-AuNPs exhibit a more intense absorbance, suggesting possible aggregation, a slight increase in particle size, or changes in the local refractive index due to cysteine binding. In the morphology of the band, we can observe a small shoulder at approximately 600 nm, only in functionalized particles, that can be related with changes in the particle’s morphology. These spectral differences, although small, may indicate chiral surface effects, which are significant in applications involving enantioselective biological interactions or chiroptical responses (e.g., circular dichroism). Any red shift or intensity change in the SPR band can affect the optical, catalytic, or biomedical behavior of the nanoparticles.

### 2.3. TEM and DLS Analysis

**Figure 3:**
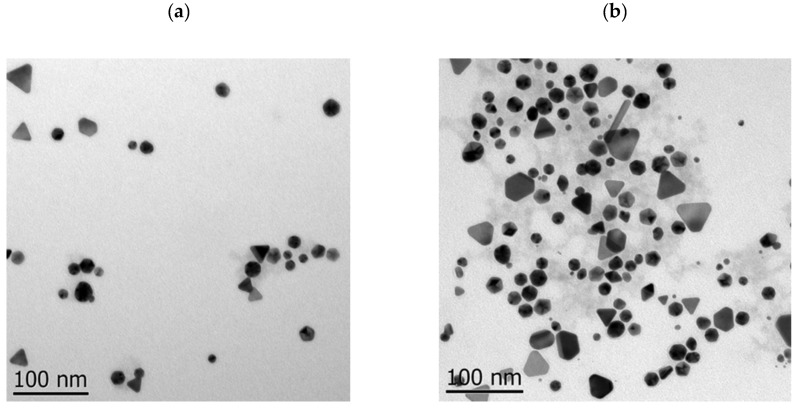

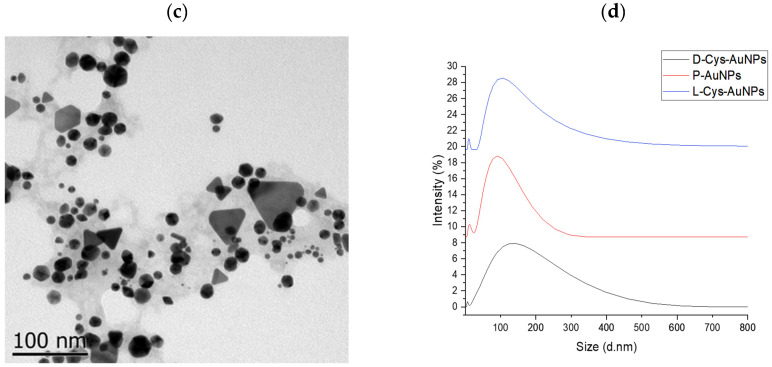
TEM micrographics of (**a**) P-AuNPs, (**b**) L-Cys-AuNPs, (**c**) D-Cys-AuNPs, and (**d**) size distribution of DLS analysis.

TEM images and DLS particle size of the P-AuNPs and L/D-Cys-AuNPs are presented in Figure 3. The particles obtained have different morphologies and sizes, with triangular and spherical morphologies predominating. The sizes in TEM micrographs are 19.8 ± 1.9, 22.6 ± 1.2, and 28.6 ± 2.3 nm for P-AuNPs, L-Cys-AuNPs, and D-Cys-AuNPs, respectively. It can be observed that modifying the particle’s surface chirality significantly increases their size, mainly with a compound of chirality D. The hydrodynamic diameter obtained by DLS analysis showed that the smallest diameters were the P-AuNPs (59.75), followed by modified L-Cys-AuNPs (91.14) and D-Cys-AuNPs (110.1). These results agree with the order observed with the STEM micrographs. However, the diameters are bigger, a fact ascribed to compounds stuck on the surface of the particles and the formation of aggregates of the particles, increasing their size.

The mechanism of functionalization with cysteine can be carried out as follows: the potential competition between SH and NH2 groups for covalent binding on the gold surface fails because the nitrogen lone pair of electrons is already protonated; after all that, the gold nanoparticles form a covalent bonding with the thiol (-SH) group. After its adsorption on the gold nanoparticles, the cysteine molecule still has two functional groups that are free to form bonds between particles, which form particle aggregates that increase the size of the particles, as can be seen in the results [25,26].

Zeta potential measures the magnitude of electrostatic (or charge) repulsion or attraction between particles and is one of the fundamental parameters known to affect stability. Particles with a zeta potential value above +30 mV (or below −30 mV) are commonly considered stable. The particles present high stability and a negative charge of −4.88 mV, −2.85 mV, and −18.6 mV for P-AuNPs, L-Cys-AuNPs, and D-Cys-AuNPs, respectively. We can observe that the change in surface chirality affects the charge quantity but not the stability [27].

Summarizing the changes observed in morphology and size after functionalization, the comparative analysis revealed that L-Cys-AuNPs were smaller (22.6 ± 1.2 nm) and more monodisperse than D-Cys-AuNPs (28.6 ± 2.3 nm), suggesting that the chirality of the surface ligand influences particle aggregation and nucleation. Additionally, in the different morphologies presented after the functionalization, the number of triangular and hexagonal particles increases. These differences are further supported by DLS measurements and zeta potential values in which the charges of the particles present a significant difference, increasing to about three times the negative charge compared to when D-Cys were used; on the contrary, when we added L-Cys, the negative charge decreased, indicating that the D-form may promote interparticle crosslinking due to different orientations of functional groups upon binding. XRD analysis confirmed that these changes were confined to surface characteristics and did not affect the core crystallinity. The rationale behind using both enantiomers lies in evaluating how surface chirality modulates physicochemical behavior and biological response. The observed trends align with previous studies reporting enantioselective interactions at the nano-bio interface, where L-amino acid ligands favor tighter packing and better colloidal stability [28,29].

### 2.4. Fourier Transform Infrared Spectroscopy (FTIR)

**Figure 4:**
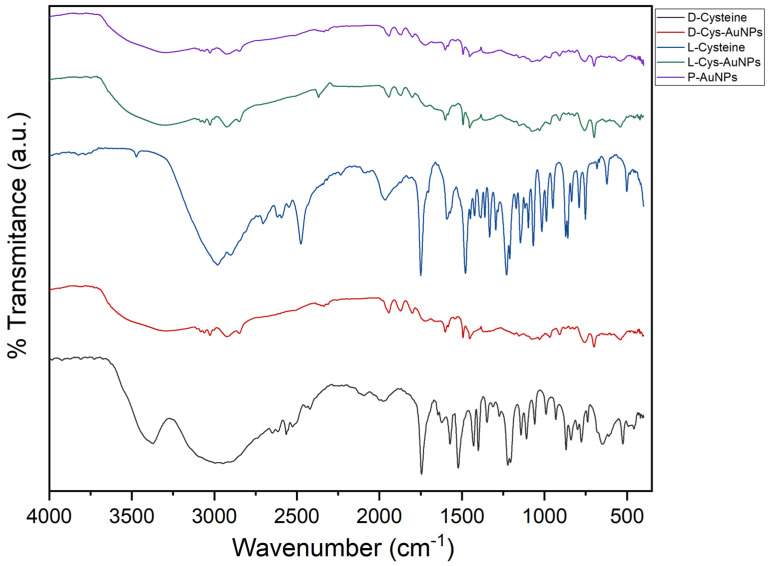
FTIR spectra of P-AuNPs, L-Cys-AuNPs, and D-Cys-AuNPs.

Functional groups involved in AuNP stabilization and surface functionalization were determined using FTIR analysis. The pure L- and D-cysteine spectra (Figure 4) showed characteristic bands for the asymmetric and symmetric stretching vibrations of -COO^−^ group at ~1590 cm^−1^ and 1400 cm^−1^, respectively. In addition, there was a weaker but well-defined band around 2520 cm^−1^, attributed to the thiol (-SH) stretching vibration, a key functional group responsible for binding to gold surfaces. In the L-Cys-AuNPs and D-Cys-AuNPs spectra, significant spectral changes confirmed the successful interaction between cysteine and the AuNP surface. The -SH stretching band (2520 cm^−1^) disappeared completely, indicating the formation of a strong Au-S bond, corresponding to the chemisorption of the thiol group onto the AuNPs surface. In addition, the carboxylate bands also showed some shifts in wavenumber and intensity, which indicated the conformational changes caused by the coordination or hydrogen bonding interactions between ligands and binding agents. The appearance of new or increased bands around 1650 cm^−1^ and 1530 cm^−1^, for the amide I (C=O stretching) and amide II (N-H bending and C-N stretching) vibrations, respectively, provides evidence of the presence of the amino acid backbone on the nanoparticle surface. These characteristics are consistent with the cysteine binds through its thiol group, while the carboxyl and amine groups remain partially exposed, contributing to the colloidal stability and potential for further functionalization. These spectral bands suggested that both L- and D-cysteine molecules were successfully added to the surface of the green-synthesized AuNPs via their thiol groups, leading to the formation of stable, chiral, and biocompatible nanostructures [30].

### 2.5. X-Ray Diffraction Analysis (XDR)

**Figure 5:**
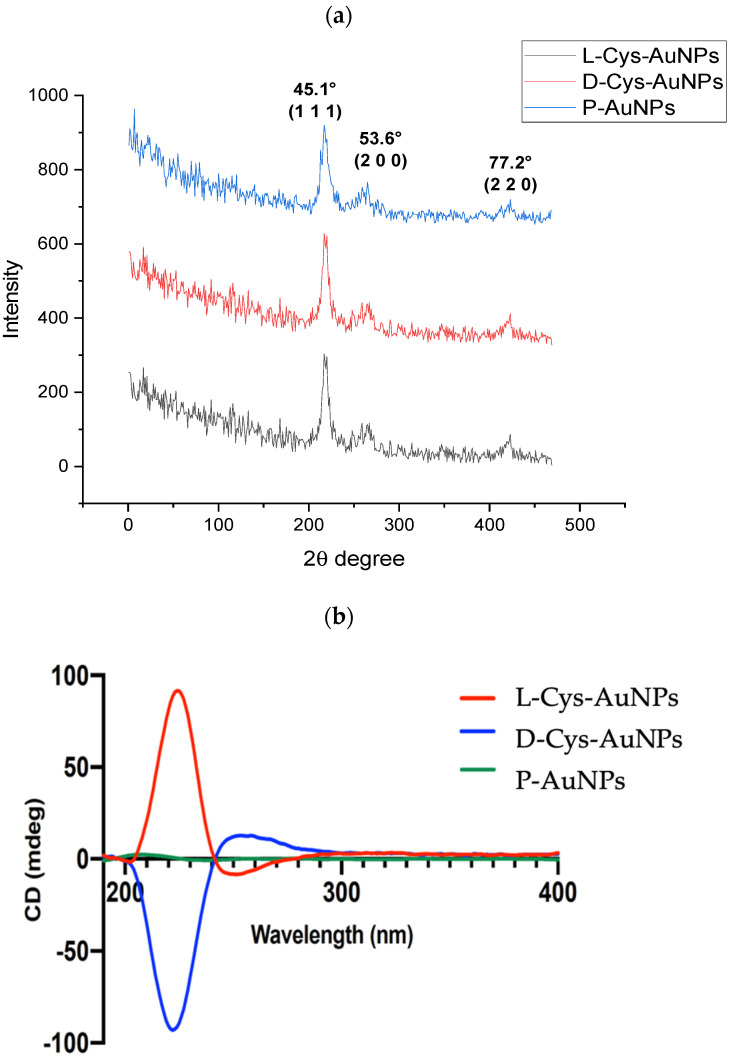
(**a**) XDR diffractograms and (**b**) ECD spectra of P-AuNPs, L-Cys-AuNPs, and D-Cys-AuNPs.

X-ray diffraction analysis was performed to determine the crystalline structure and phase purity of the gold nanoparticles synthesized with *S. molle* extract and subsequently functionalized with L- and D-cysteine. As shown in Figure 5a, all samples showed four dominant diffraction peaks at 2θ values of approximately 38.2°, 44.4°, 64.6°, and 77.5°, which could be well indexed to the (1 1 1), (2 0 0), (2 2 0), and (3 1 1) planes of face-centered cubic (fcc) metallic gold, according to the JCPDS card No. 04-0784. The strong intensity of the (111) lattice plane suggests a preferred orientation along this plane, typically observed in colloidal AuNPs synthesized under mild conditions. This preferential growth is typically associated with enhanced stability and catalytic performance due to the high atomic density of the (111) facet [31,32]. Notably, there were no other peaks observed, indicating that there were no gold oxides or residual crystalline components from the plant extract or capping agents. This result demonstrates the high phase purity of the synthesized nanoparticles [33].

The average crystallite size was estimated using the Debye–Scherrer equation based on the full width at half maximum (FWHM) of the (111) peak. The size of each sample was found to be between 12 and 18 nm and there was no notable change upon surface modification with cysteine. These findings imply that the crystalline core of the nanoparticles is not modified upon functionalization. Altogether, the XRD pattern confirms the successful synthesis of highly crystalline, monometallic AuNPs with fcc structure. Moreover, the preservation of crystallinity and size after functionalization supports the stability of the nanostructures and validates the gentle, green synthesis route used in this study [34].

### 2.6. Circular Dichroism (CD) Analysis

Circular dichroism (CD) spectroscopy was employed to investigate the chiroptical properties of gold nanoparticles (AuNPs) functionalized with L- and D-cysteine. As shown in Figure 5b, a significant difference in the CD spectra was observed between L-Cys-AuNPs and D-Cys-AuNPs, whereas the non-functionalized nanoparticles (P-AuNPs) showed no detectable CD signal in the range of 190–400 nm, confirming their achiral nature.

The L-Cys-AuNPs displayed a strong positive Cotton effect at approximately 215 nm, followed by a negative band near 230 nm, while the D-Cys-AuNPs showed an inverted profile with a negative peak around 215 nm and a positive band near 230 nm. These mirror-image spectra are indicative of the successful transfer of molecular chirality from the enantiomeric cysteine molecules to the gold nanoparticle surface. This behavior is attributed to electronic transitions, primarily n → π* and π → π*, involving the amide and carboxyl groups of cysteine when adsorbed onto a metallic chiral surface [9,35,36].

These findings are consistent with previous reports where chiral ligands, such as amino acids or thiol-containing enantiomers, induced optical activity in plasmonic and non-plasmonic metal nanoparticles [37,38]. The chiral signals further support the formation of strong Au-S bonds between the cysteine thiol group and the nanoparticle surface, resulting in a stable chiral environment that can modify the electronic distribution at the metal interface [39]. Such chiral surface environments are of particular interest for applications in enantioselective catalysis, biosensing, and chiral recognition technologies [40].

### 2.7. Antimicrobial Activity

The antimicrobial activity of the *Schinus molle* aqueous extract, green gold nanoparticles (P-AuNPs), and L-/D-cysteine-functionalized AuNPs were tested against a representative series of clinically relevant microorganisms, including Gram-negative (*Escherichia coli*, *Salmonella enterica*), Gram-positive (*Listeria monocytogenes*, *Staphylococcus aureus*, *Staphylococcus epidermidis*), and an opportunistic fungal pathogen *Candida albicans* (Table 1).

As would be expected, the *S. molle* extract exhibited moderate antibacterial activity at higher concentrations, and this was more apparent for the Gram-positive strains. The phytochemical content rich in phenolics, flavonoids, and tannins contributes to its antimicrobial profile, as previously reported for other extracts of this species [13]. However, its antifungal effect against *C. albicans* was modest (18 ± 0.0 mm).

Functionalization of the green-synthesized AuNPs with enantiomers of cysteine notably enhanced their antimicrobial spectrum and potency. The L-Cys-AuNPs demonstrated the strongest and broadest antimicrobial activity, with inhibition zones up to 32.0 ± 1.0 mm against *S. epidermidis* and remarkable efficacy against *C. albicans* (23.0 ± 0.0 mm). In contrast, D-Cys-AuNPs showed moderate antibacterial activity and no inhibition of *C. albicans*. The significant inhibition of *C. albicans* by L-Cys-AuNPs is of particular interest, as fungal infections caused by this opportunistic pathogen are increasingly prevalent and often difficult to treat due to biofilm formation and antifungal resistance [36,37]. P-AuNPs did not exhibit antifungal activity, indicating that the antifungal effect is not related to the presence of nanoparticles but likely a result of synergistic interactions between AuNPs and the L-cysteine functionalization. L-cysteine, containing a thiol (-SH) and amine group, may enhance interaction with fungal membranes, disrupt metabolic processes, and facilitate ROS generation. Additionally, chirality appears to play a key role; the superior performance of L-Cys-AuNPs compared to their D-counterpart suggests that enantioselective interactions with microbial targets may enhance bioactivity, as previously described in chiral nanomaterial studies [36,37]. These findings highlight the potential of chiral surface engineering to tailor nanomaterials for specific antimicrobial applications, especially in the context of resistant fungal pathogens like *Candida albicans*. The development of such nanoformulations could represent a valuable strategy in next-generation antimicrobial therapies.

The MIC and MBC values for P-AuNPs, D-Cys-AuNPs, and L-Cys-AuNPs against the selected panel of microorganisms are summarized in Table 2. These values further corroborate the findings from the agar diffusion tests and provide deeper insights into the bacteriostatic and bactericidal potentials of the synthesized nanomaterials. L-Cys-AuNPs exhibited the lowest MIC values across all tested organisms, ranging from 0.25 mg/mL for bacteria to 1.25 mg/mL for *C. albicans*, indicating high potency even at low concentrations. Moreover, their MBC values, particularly 1:4 and 1:8 dilutions, suggest a strong bactericidal effect, especially against *L. monocytogenes* and *S. enterica*. In contrast, P-AuNPs and D-Cys-AuNPs required higher concentrations (up to 5.0 mg/mL) to achieve comparable effects. For example, the MIC for E. coli was 0.42 mg/mL for P-AuNPs, 0.36 mg/mL for D-Cys-AuNPs, but only 0.25 mg/mL for L-Cys-AuNPs, while the MBC dropped to 1:4 for L-Cys-AuNPs, a fourfold reduction compared to the controls. To contextualize the antifungal performance of L-Cys-AuNPs, we compared their efficacy with commonly used antifungal agents such as fluconazole and amphotericin B, based on the literature-reported MIC. While fluconazole typically has an MIC around 0.75 μg/mL and amphotericin B has an MIC of 1.0 μg/mL, L-Cys-AuNPs produced comparable concentrations (1.25 mg/mL), highlighting their promising therapeutic potential. This suggests that L-Cys-AuNPs could serve not only as standalone antifungal agents but also as candidates for combination therapy in treating drug-resistant strains [42].

Importantly, *C. albicans*, a common cause of systemic fungal infections, showed resistance to P-AuNPs and D-Cys-AuNPs, which failed to demonstrate fungicidal activity at the tested concentrations. In contrast, L-Cys-AuNPs achieved an MIC of 1.25 mg/mL and an MBC of 2.5 mg/mL, highlighting their unique antifungal capability. This finding is especially relevant in the context of growing antifungal resistance and the limited availability of effective antifungal agents [34]. The efficacy of L-Cys-AuNPs against *C. albicans* may be attributed to the chiral interface and enhanced interaction with fungal membranes, promoting ROS generation, membrane disruption, and interference with intracellular pathways; another important characteristic is the size of the gold particles, as well as the significant increase in negative charges on the surface, which does not allow adequate interaction with the cell wall of the microorganisms. Although direct mechanistic assays were not conducted in this study, the previous literature supports the involvement of oxidative stress, mitochondrial dysfunction, and cell wall destabilization in similar systems. Further work using ROS quantification, mitochondrial membrane potential analysis, and membrane integrity assays is warranted to elucidate the dominant mechanism of action [37,38].

The significantly lower MIC and MBC values observed for L-Cys-AuNPs compared to D-Cys-AuNPs and unmodified P-AuNPs suggest that chirality at the nanoparticle surface plays a key role in enhancing antimicrobial efficacy. This enhanced activity may arise from stronger enantioselective interactions between L-cysteine-coated nanoparticles and microbial cell membranes. It is essential to note that the antimicrobial activity of gold nanoparticles (AuNPs) is strongly influenced by their size and surface charge. Smaller AuNPs have a greater ability to penetrate microbial cell membranes, facilitating the intracellular generation of reactive oxygen species (ROS). These ROS can damage vital organelles, such as mitochondria, ultimately leading to cell death. In addition, the surface charge, typically assessed through zeta potential measurements, plays a crucial role in nanoparticle–cell interactions. Negatively charged AuNPs generally exhibit greater colloidal stability due to electrostatic repulsion; however, this same repulsion can hinder their adhesion to negatively charged bacterial cell membranes, potentially reducing their antimicrobial efficacy. Therefore, optimizing both the size and surface charge of AuNPs is essential to enhance their biological activity while maintaining stability in biological environments. In this sense, the L-Cys-AuNPs exhibit a lower size than D-Cys-AuNPs and a lower negative surface charge, which can be related to their greater antifungal and antimicrobial activity [43,44]. These results position L-Cys-AuNPs as a promising nanomaterial for broad-spectrum antimicrobial applications, with particular potential in combating fungal infections and drug-resistant bacteria.

## 3. Materials and Methods

### 3.1. Chemicals and Reagents

Solvents, chloroauric acid (HAuCl4), sodium, 1,1-diphenyl-2-picrylhydrazyl (DPPH), 2,2′-azinobis-(3-ethylbenzothiazoline-6-sulfonic acid) (ABTS), and potassium persulfate were purchased from Sigma Chemical Co. (St. Louis, MO, USA).

### 3.2. Extraction and Characterization

Molle (*Schinus molle*) (World Checklist of Selected Plant Families, WCSP record in review: Sp. Pl.: 389.) fruits were collected in Hidalgo (Mexico) and deposited in the “Dr. Jerzy Rzedowski” (QMEX) herbarium of Universidad Autónoma de Querétaro (Juriquilla, Querétaro, Mexico). The selected fruits were washed with deionized water and dried in a convection oven at 60 °C for 6 h. The dried residues were then powdered to fine particles using a mixer grinder GX4100 (KRUPS, Solingen, Germany) and passed through a 60-mesh screen (250 µm particle size). The Ultrasonic-Assisted Extraction (UAE) was performed with the methodology described by Gonzales-Silva et al., 2022, with some modifications [45]: First, 10 g of ground Pirul seed was placed in a 250 mL beaker and extracted with 100 mL distilled water using an ultrasonic probe UP400S (Hielscher ultrasonics, Teltow, Germany). The sonicator tip was immersed 30 mm into the sample at an amplitude of 50% for 15 min. The ultrasonication extraction process was carried out in ice water. The extracts were filtered (Whatman Paper no. 40, Whatman plc, Kent, UK) and stored at 4 °C for further analysis. To spectrophotometrically analyze phenolic compounds, total phenolic compounds (TPC) were determined using the Folin–Ciocalteu technique, and total phenolics were expressed as mg of gallic acid equivalents per gram of dry weight (mg GAE/g DW). For the in vitro antioxidant capacity (AOX), the DPPH [46] and ABTS^+^ [47] radicals scavenge were tested, and the results were indicated as mg Trolox equivalents per gram of dried weight (mg TE/g DW).

### 3.3. Green Synthesis of Gold Nanoparticles with Pirul SEED Extract

For the green synthesis of PAuNPs, we used the method reported previously [2]: First, 10 mL of an aqueous solution of HAuCl_4_ (0.5 mM) was mixed with 100 μL of the aqueous extract of Pirul seeds (P-AuNPs). The reaction mixture was incubated at 60 °C for 5 min; the reduction of Au^3+^ ions was monitored using color changes, and the UV−Vis spectrum was recorded from 300 to 1000 nm with baseline correction using water as the blank on a Agilent 8453 UV–Visible Spectrophotometer (Santa Clara, CA, USA).

### 3.4. Synthesis of L/D-Cys-AuNPs

The L/D-Cys-AuNPs were prepared with the methodology described by Zhang et al., 2021 [28], with some modifications. First, 0.1 mL of 1 × 10^−4^ M of L or D-cysteine solutions was added to 5 mL of the green AuNP solution, followed by 2 h of stirring, before centrifuging the solutions. After centrifugation, the supernatants were removed. The precipitates were dispersed in deionized water by sonication.

### 3.5. AuNPs Characterization

The morphology and diameters of AuNPs were determined using a scanning transmission electron microscope (STEM SU8230, Hitachi, Tokyo, Japan). The diameters of the AuNPs were obtained by measuring at least 50 particles with Image J ^®^ 1.43 software (National Institutes of Health and the Laboratory for Optical and Computational Instrumentation, Madison, WI, USA) according to Chai et al., 2010 [34]. Infrared absorption spectra of dried AuNPs and lyophilized extracts were recorded in Fourier-transformed infrared (FT-IR) equipment (Spectrometer Spectrum Two, PerkinElmer, Shelton, CT, USA); this method used Diffuse Reflectance, 24 scans, and 4 cm^−1^ resolution. The X-ray pattern of the AuNPs was obtained with an X-ray diffractometer (Dmax 2100 Rigaku Americas Corporation, The Woodlands, TX, USA) that has a cobalt emission line (k = 1.7889º A), from 5 to 50º, on a 2θ scale, with a step size of 0.02º. The circular spectra were obtained using a J-1500 spectropolarimeter instrument (JASCO, Tokyo, Japan). The CD spectrum of each sample condition was measured in the forwards and backwards directions by changing the direction of the sample relative to the incident light [48].

### 3.6. Antimicrobial Activity

#### 3.6.1. Pathogens Strains

Six microbial strains were selected to evaluate the antimicrobial activity: five bacterial pathogens (*Escherichia coli* ATCC1129, *Salmonella enterica* Typhimurium ATCC 14028, *Listeria monocytogenes* ATCC 19115, *Staphylococcus aureus* ATCC 6538, *Staphylococcus epidermidis* ATCC 12228) and one yeast (*Candida albicans* ATCC 10231). These strains were obtained from the Food Microbiological Risk Assessment and Control Laboratory at the Autonomous University of Queretaro, Mexico. Strains were preserved in trypticase soy broth with 15% glycerol at −80 °C. For activation, stock cultures were streaked on blood agar base slants and incubated at 35 °C for 24 h.

#### 3.6.2. Antimicrobial Susceptibility Testing

The antimicrobial activity and susceptibility of all strains were evaluated using standardized Clinical and Laboratory Standards Institute (CLSI) protocols. Bacterial susceptibility assays employed Mueller–Hinton broth and agar as recommended by CLSI. Due to the unavailability of CLSI-recommended media for *Candida albicans*, susceptibility was assessed using Mueller–Hinton broth and Mueller–Hinton agar supplemented with 2% glucose as substitute media. Prior growth controls confirmed adequate development of *C. albicans* in these media, validating the antimicrobial testing under these adapted conditions.

#### 3.6.3. Well-Diffusion Antimicrobial Analysis

Stock cultures were streaked onto trypticase soy agar (TSA) plates and incubated at 35 °C for 24 h. Colonies were harvested and then suspended in sterile saline to achieve a turbidity equivalent to a 0.5 McFarland standard. Following CLSI guidelines, suspensions were evenly spread onto Mueller–Hinton agar plates [49]. Four wells (8 mm diameter) were aseptically created in each plate and filled with 50 µL of various concentrations (10, 5, 2.5, 1.25, 1, and 0.1 mg/mL) of *Schinus molle* extract, *Schinus molle*-based nanoparticles, and cysteine-functionalized nanoparticles. Plates were incubated at 35 °C for 24 h, after which inhibition zones were measured.

#### 3.6.4. Minimum Inhibitory Concentration (MIC) and Minimum Bactericidal Concentration (MBC) of AuNPs

MIC was determined by broth microdilution as described in CLSI guidelines [50]. Each well of a sterile 96-well microplate was inoculated with 100 µL of Mueller–Hinton broth containing approximately 1 × 10^6^ CFU/mL of the test microorganism, along with 100 µL of nanoparticle suspensions at concentrations ranging from 5 to 0.05 mg/mL. Plates were incubated aerobically at 35 °C for 24 h. MIC was defined as the lowest concentration of an antimicrobial agent that inhibits visible microbial growth. To determine MBC, 10 µL aliquots from wells with no visible growth were plated onto TSA and incubated at 37 °C for 24 h. MBC was defined as the lowest concentration resulting in a ≥99.9% reduction in the initial inoculum, indicated by the absence of colony growth.

## 4. Conclusions

The aqueous extract of *Schinus molle* (Pirul) fruits demonstrated a high content of phenolic compounds, particularly simple acids such as gallic acid, which played a key role as reducing agents in the green synthesis of gold nanoparticles (AuNPs), allowing the formation of AuNPs without the need for additional stabilizers or toxic reagents, making this the first report of the use of Pirul extract for the synthesis of metallic nanoparticles. The resulting nanoparticles exhibited diverse morphologies, including quasi-spherical and triangular shapes. These nanoparticles were successfully functionalized with cysteine enantiomers (L- and D-cysteine), marking the first time that green-synthesized AuNPs have been surface-engineered with chiral amino acids. This modification induced significant morphological and physicochemical changes. Notably, functionalization with D-cysteine led to larger particle sizes, likely due to aggregation, as well as a substantial shift in zeta potential, indicating a change in surface charge. These modifications were correlated with a reduction in antimicrobial activity and a more selective interaction with certain microorganisms. In contrast, functionalization with L-cysteine conferred potent antifungal properties to the AuNPs, particularly against *Candida albicans*, a pathogen of major medical concern. This selective antimicrobial strategy highlights the influence of chirality in tailoring the biological activity of nanoparticles and offers valuable insights for designing novel chiral antimicrobial agents with enhanced efficacy and biocompatibility. These findings underscore the broader significance of chirality in nanomedicine. The enantioselective enhancement observed with L-Cys-AuNPs suggests that chirality can be a powerful design parameter in developing next-generation antimicrobial nanomaterials. Leveraging chiral interactions may allow for more targeted, potent, and selective disruption of microbial cells, especially in the context of multidrug-resistant fungi like *C. albicans*. Future research should explore the stereospecific recognition and binding phenomena at play between chiral nanostructures and biological targets.

## Figures and Tables

**Table 1:** Inhibition zone diameter of *Schinus molle* extract and functionalized particles against pathogenic bacteria and yeast. These results were presented at the Ninth International Symposium on Dielectric Materials and Applications (ISyDMA’9) and published in the proceedings [41].

Antimicrobial Suspension		Microorganisms					
		*E. coli*	*S. enterica*	*L. monocytogenes*	*S. aureus*	*S. epidermidis*	*C. albicans*
*Schinus molle* extract	10 mg/mL	11.7 ± 0.6	14.3 ± 0.6	17.0 ± 1.7	17.7 ± 1.5	17.7 ± 0.6	PI (18 ± 0.0)
	5 mg/mL	NI	NI	13.3 ± 1.5	13.7 ± 1.5	14.0 ± 1.0	PI (10.7 ± 0.6)
	2.5 mg/mL	NI	NI	12.3 ± 0.6	13.0 ± 1.0	12.0 ± 0.0	NI
	1.25 mg/mL	NI	NI	NI	9.7 ± 0.6	NI	NI
	0.1 mg/mL	NI	NI	NI	9.0 ± 0.0	NI	NI
P-AuNPs	10 mg/mL	21 ± 1.0	22.7 ± 1.5	19.7 ± 0.6	25.7 ± 1.5	26.7 ± 0.6	NI
	5 mg/mL	12.3 ± 1.5	16.7 ± 1.2	NI	13.0 ± 1.0	9.3 ± 0.6	NI
	2.5 mg/mL	NI	NI	NI	10 ± 0.0	NI	NI
	1.25 mg/mL	NI	NI	NI	NI	NI	NI
	0.1 mg/mL	NI	NI	NI	NI	NI	NI
D-Cys-AuNPs	10 mg/mL	15.7 ± 1.2	17.0 ± 1.7	14.7 ± 1.5	21.3 ± 1.7	21.0 ± 1.7	NI
	5 mg/mL	9.7 ± 0.6	10.7 ± 1.2	NI	15.7 ± 1.2	9.3 ± 0.6	NI
	2.5 mg/mL	NI	NI	NI	12.7 ± 0.6	NI	NI
	1.25 mg/mL	NI	NI	NI	NI	NI	NI
	0.1 mg/mL	NI	NI	NI	NI	NI	NI
L-Cys-AuNPs	10 mg/mL	24.0 ± 1.0	25.3 ± 0.6	26.3 ± 0.6	28.0 ± 1.0	32.0 ± 1.0	PI (23.0 ± 0.0)
	5 mg/mL	18.7 ± 1.5	22.0 ± 1.0	16.3 ± 0.6	20.3 ± 0.6	18.0 ± 0.0	PI (10.7 ± 0.6)
	2.5 mg/mL	13 ± 1.0	15.0 ± 1.0	NI	15.7 ± 0.6	13.3 ± 0.6	NI
	1.25 mg/mL	NI	11.0 ± 1.0	NI	13.7 ± 0.6	11.3 ± 0.6	NI
	0.1 mg/mL	NI	10.0 ± 1.0	NI	11.3 ± 0.6	NI	NI

(NI: non-inhibition, PI: partial inhibition, some colony growth inside inhibition halo).

**Table 2:** MIC and MBC of nanoparticles of P-AuNPs, D-Cys-AuNPs, and L-Cys-AuNPs.

Microorganisms	P-AuNPs		D-Cys-AuNPs		L-Cys-AuNPs	
	MIC (mg/mL)	MBC (mg/mL)	MIC (mg/mL)	MBC (mg/mL)	MIC (mg/mL)	MBC (mg/mL)
*E. coli*	0.42	5.0	0.36	5.0	0.25	1.4
*S. enterica*	0.42	5.0	0.36	2.5	0.25	1.4
*L. monocytogenes*	0.50	5.0	0.42	5.0	0.25	1.8
*S. aureus*	0.50	--	0.42	5.0	0.25	5.0
*S. epidermidis*	0.50	5.0	0.36	5.0	0.25	2.5
*C. albicans*	2.5	--	2.5	--	1.25	2.5

## Data Availability

The original contributions presented in this study are included in the article. Further inquiries can be directed to the corresponding authors.
